# Continuous Erector Spinae and Serratus-Intercostal Block With Ketamine-Dexmedetomidine Sedation for Quadrantectomy and Axillary Dissection in a Multimorbid Patient

**DOI:** 10.7759/cureus.45071

**Published:** 2023-09-11

**Authors:** Massimiliano Luca D'Agostino, Paolo Scimia, Antonio De Cato, Marta Muscelli, Chiara Angeletti

**Affiliations:** 1 Department of Life, Health & Environmental Sciences (MeSVA), San Salvatore Teaching Hospital of L’Aquila, University of L'Aquila, L'Aquila, ITA; 2 Operative Unit of Anaesthesiology, Intensive Care and Pain Medicine, Civil Hospital G. Mazzini, Teramo, ITA; 3 Department of Life, Health & Environmental Sciences (MeSVA), San Salvatore Teaching Hospital of L’Aquila, University of L'Aquila, L’Aquila, ITA

**Keywords:** opioid-free analgesia, dexmedetomidine, general surgery and breast cancer, serratus plane block, serratus anterior plane (sap) block, opioid free anesthesia dexdor in anesthesia, esp block, multimorbility, ketamine-dexmedetomidine, erector spinae plane (esp) block

## Abstract

Multimorbidity is a clinical presentation that poses an increased risk of perioperative and postoperative complications. Tailored anaesthetic management could potentially minimise the risk of negative outcomes.

Peripheral nerve and fasciae blocks are valid strategies for perioperative and postoperative pain management, which avoid complications related to general anaesthesia and reduce the risk of intensive care unit admission as well as the hospital length of stay.

We describe the case of a 56-old patient with multimorbidity, including obesity with a BMI of 45.7, unstable angina, predicted difficult airway management and obstructive sleep apnoea syndrome (OSAS) scheduled for left mastectomy with sentinel lymph node biopsy, managed with a left continuous thoracic erector spinae plane (ESP) block plus serratus-intercostal plane block (BRanches of Intercostal nerves at the Level of Mid-Axillary line (BRILMA)), and sedation with combined ketamine-dexmedetomidine.

Fascial blocks combined with opioid-free anaesthesia (OFA) proved to be effective for the multimorbid patient, ensuring successful perioperative management and a proper recovery after surgery.

## Introduction

Multimorbidity is defined as a clinical condition resulting in the coexistence of two or more chronic or acute diseases [1]. This leads to an increased perioperative risk and vulnerability to potential postoperative complications [1,2].

According to Cavalli and colleagues [3], the preoperative assessment of associated comorbidity risk is necessary for surgical procedure management.

For better management of both intraoperative anaesthesia and postoperative outcomes, is preferred the practice of peripheral nerve blocks (PNB) for patients with multimorbidity and obesity-related complications. [4]

Considering this, regional anaesthesia (RA) can represent a valid strategy for managing perioperative-relevant multimorbidity in surgical patients. Regional anaesthesia prevents some of the complications associated with general anaesthesia (GA) such as airway problems, challenges of mechanical ventilation, and cardiovascular depression. It reduces both the postoperative pain and the risk of intensive care unit (ICU) stay and hospitalization. Regional anaesthesia minimises the alteration of physiological function while providing analgesia without compromising consciousness. 

According to previous studies [5], the introduction of the ultrasound-guided erector spinae plane block (Us-ESPB) in breast surgery can represent an alternative to GA and more extensive locoregional techniques (i.e., epidural anaesthesia, paravertebral block), especially in multimorbidity patients [6]. Based on recent results, this innovative interfascial plane technique, as well as blocking BRanches of Intercostal nerves at the Level of Mid-Axillary line (BRILMA block), seems to play a pivotal role in managing analgesia in breast surgery [7].

BRILMA plus erector spinae plane (ESP) offers greater guarantees of coverage in the axillary area since the operation could have involved lymph node dissection. This is because ESP has significant variability in terms of the dermatomal block extension [8].

We present the case of a multimorbid male patient undergoing mastectomy surgery for breast cancer managed through the combination of continuous ESPB and BRILMA, in sedation with ketodex.

An Opioid Free Anesthesia (OFA) with analgo-sedation in spontaneous breathing guaranteed by intravenous administration of ketamine (keta) and dexmedetomidine (dex) mixture was chosen as a personalized approach.

## Case presentation

**Figure 1:**
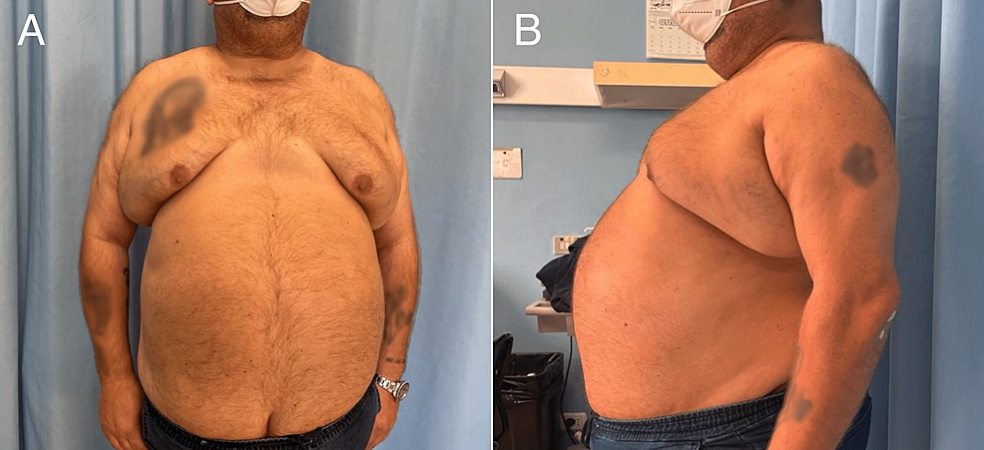
The patient's physical characteristics on preoperative assessment.

We describe a case of a 56-year-old male patient (Figure 1A, 1B) with multiple morbidities (Table 1) who underwent to left lower external quadrantectomy prior scheduled for left mastectomy with sentinel lymph node biopsy for suspected breast cancer.

**Table 1:** Summary of the patient’s medical history BMI: body mass index; COPD: chronic obstructive pulmonary disease; NSTEMI: non-ST-segment elevation myocardial infarction; OSAS: obstructive sleep apnoea syndrome; CPAP: continuous positive airway pressure

MEDICAL HISTORY
Third-class obesity (BMI 45.7; weight 140 kg, height 175 cm)
COPD
Sarcoidosis
Hypothyroidism
Diabetes mellitus type 2
Familiar hypercholesterolaemia
Gout
Metabolic syndrome
Ailments of the lacrimal apparatus
Hypertension
Unstable angina
Acute myocardial stroke NSTEMI
Transient ischemic attack
Phlebitis
Right popliteal vein thrombosis
Bilateral interstitial pneumonia from SARS-CoV-2
Gastroesophageal reflux disease
Bilateral carpal tunnel syndrome
OSAS
CPAP mask night and day
Chronic renal failure
Proteinuria

The patient had an American Society of Anesthesiologists (ASA) status of 4, and during the anesthesiology visit, he reported angina and exertional dyspnea. He underwent coronary angioplasty in 2010 and three coronary artery bypass grafts in November 2012. The cardiologist classified the patient as “elevated risk”. Spirometry showed a mild restrictive ventilatory deficit, and the pulmonologist indicated the use of a continuous positive airway pressure (CPAP) mask on waking if general anaesthesia was administered.

During the perioperative evaluation, the patient was thoroughly informed about the risks and benefits of the proposed locoregional anaesthetic techniques and about the alternative procedures that would have been implemented if these techniques were unsuccessful.

For the preoperative phase, after premedication with midazolam 2 mg, an ultrasound-guided left continuous thoracic ESPB (T5) was performed with the patient in a sitting position.

**Figure 2:**
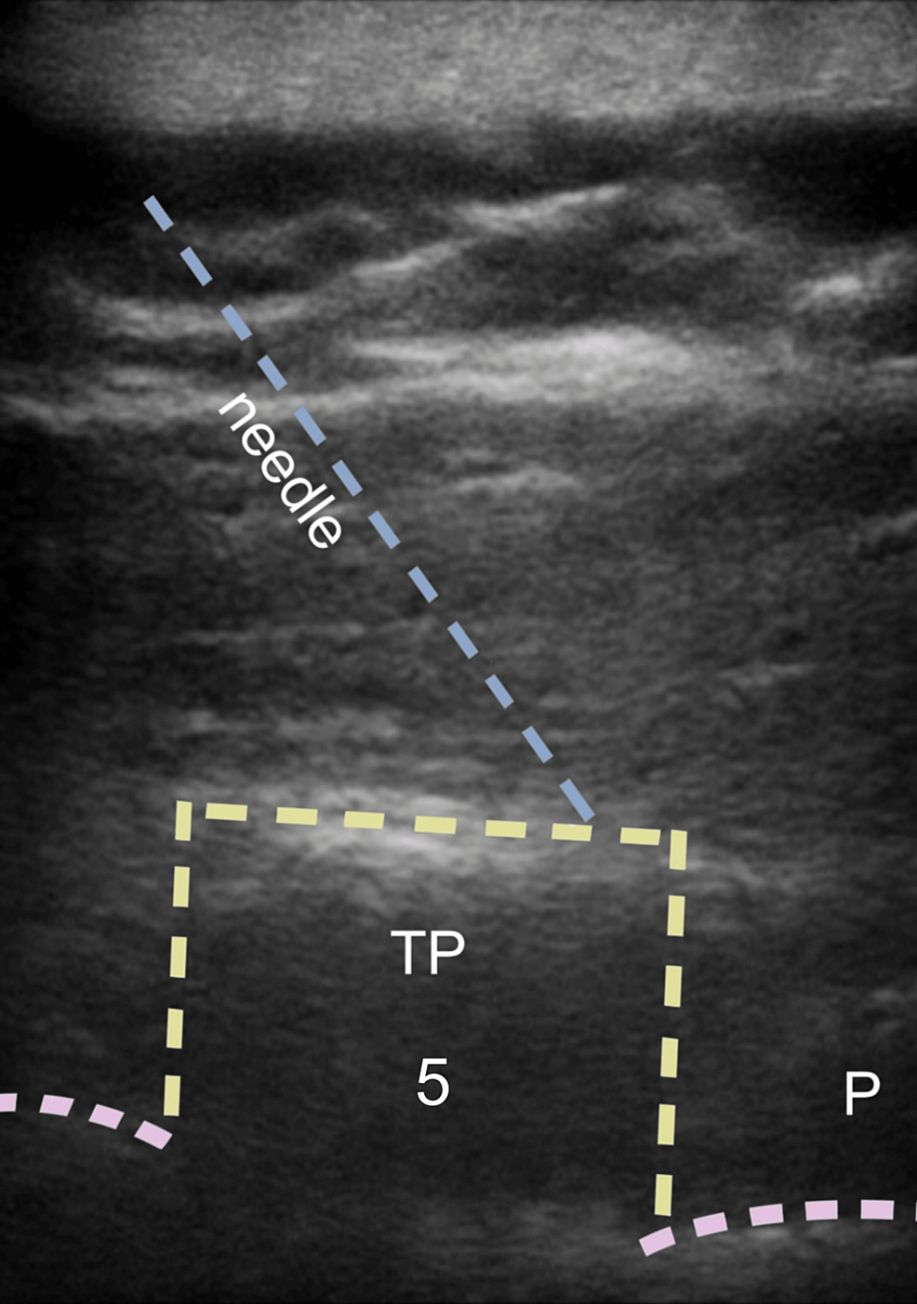
Erector spinae plane-block TP: transverse process; P: pleura

A high-frequency linear probe with longitudinal orientation (13-6 MHz, Sonosite Europe, Amsterdam, Netherlands) was placed approximately 3 cm from the midline to identify the deep plane of the erector spinae muscle (Figure 2).

**Figure 3:**
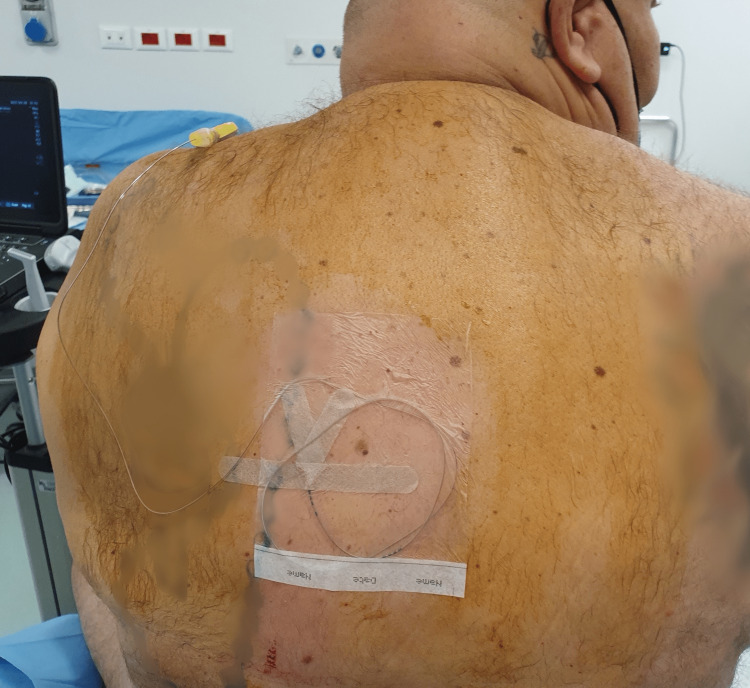
Catheter placement Right after the injection of anaesthetics in the target fascial plane, a catheter was placed 3.5 cm from the Tuohy needle tip and then secured on the patient’s back.

**Figure 4:**
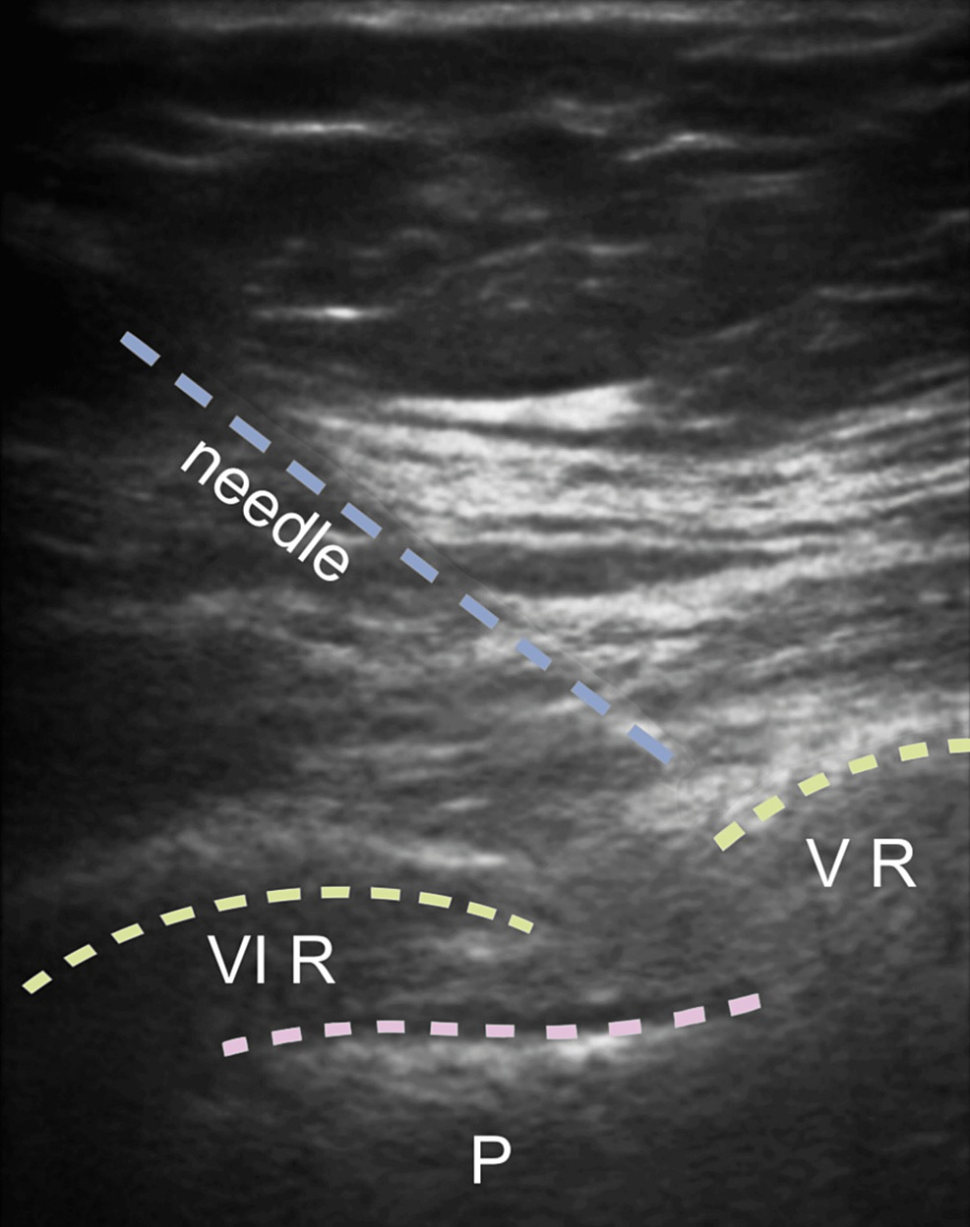
Anesthetic serratus-intercostal plane-block (BRILMA) VI R: 6th rib; V R: 5th rib; P: pleura; BRILMA: BRanches of Intercostal nerves at the Level of Mid-Axillary line

During the procedure, a Tuohy needle was inserted using a craniocaudal direction and an in-plane approach. An anaesthetic solution was injected into the target fascial plane, consisting of 20 ml of levobupivacaine 0.5% (100 mg), 50 mcg of dexmedetomidine, and 10 ml of mepivacaine 0.5% (50 mg). The catheter was then placed 3.5 cm from the needle tip (Figure 3). Following this, a left BRILMA block was performed with the patient positioned in the right lateral decubitus position. A 22 G echogenic needle was used with an in-plane approach, and 20 ml of levobupivacaine 0.5% (100 mg) and 50 mcg of dexmedetomidine were injected into the fascial plane (Figure 4).

Sedation was induced with a 1 mcg/kg bolus of dexmedetomidine over 20 minutes (total dose 140 mcg). The pinprick test performed 30 minutes after the fascial block yielded negative results. Sedation was maintained with a continuous infusion of 0.4-0.5 mcg/kg/h of dexmedetomidine, and a starting dose of 0.5 mg/kg of ketamine (70 mg). After 60 minutes, an additional 30 mg of ketamine was administered. Standard intraoperative monitoring was used, including BIS (bispectral index range: 60-90) and nasal cannulae with capnography. An oxygen flow of 3 L/min was administered.

After the nodule isolation, the histological findings indicated that the nodule was not malignant. A lower external quadrantectomy and axillary lymph node biopsy were performed, and the sample was sent to the laboratory for classification. No hypotension was recorded during the 120-minute surgical procedure, and the Ramsey Sedation Scale (RASS) was 3.

The infusion of dexmedetomidine was stopped at the end of the surgery, and the patient experienced a rapid awakening. He maintained stable vital parameters, good pain control (NRS=0), and did not experience postoperative nausea and vomiting (PONV). No pre-emptive intraoperative and postoperative systemic analgesic therapy was used. Levobupivacaine 0.125% (total volume 40 ml, rate 5 ml/h) was administered with an elastomeric pump, and the catheter was removed 8.5 hours later.

Post-operative pain evaluation at 3-6-12-24 and 48 hours in static (in bed, supine/sitting position) and dynamic (at the side/standing mobilization) conditions revealed a substantial absence of pain. After 12 hours, NRS = 3 in dynamic component was recorded and treated with Paracetamol 1 g i.v. The patient was promptly mobilized, and the postoperative course was free from complications. The patient reported satisfaction and comfort with the anesthetic technique in the postoperative period. They went back home during the second postoperative day.

Six months after discharge, the patient was interviewed, and there was no evidence of chronic pain or infectious complications. Moreover, the patient confirmed high satisfaction with the perioperative procedures performed. They returned to everyday work and daily life activities. Written informed consent for publication was obtained.

## Discussion

Multimorbidity refers to the simultaneous occurrence of multiple diseases in an individual. This condition is frequently linked to reduced quality of life, particularly in older individuals, and poses a significant challenge for healthcare professionals, including clinicians, surgeons, and anesthesiologists. Given the increased risk that such patients face during surgery, it is recommended that minimally invasive anaesthesia be used in such cases [9]. The primary objectives of preoperative medical assessments are to minimize the risk of surgical and anaesthetic complications and enable patients to return to their desired physical state as quickly as possible [10].

Prior to making an incision, the process of surgical recovery begins with a thorough preoperative evaluation. This evaluation plays a crucial role in impacting a patient's surgical stress response and postoperative outcomes [11]. While it is always recommended to carefully evaluate patients before surgery, in certain cases, an OFA approach may be appropriate. This approach can involve the use of fascial plane blocks and sedation with a combination of ketamine and dexmedetomidine. Based on our previous experience, utilizing multiple blocks on the same anatomical target can result in improved anaesthetic coverage, even for patients who are obese or present with difficult airway management [7]. If difficult airways are anticipated, RA may be considered as a preferred technique, if all safety standards outlined in the PASS checklist flowchart are strictly adhered to [12].

ESP block usually covers the whole hemithorax, but the anaesthetic solution spread is unpredictable; especially for the median line, the anaesthetic coverage may be not reached. As demonstrated by Selvi O et al. [13], cutaneous erector spinae plane sensory block at the T9 vertebral level revealed variable results and low failure rates and varying results. The efficacy of the ESP block in covering the axillary region is still a topic of debate in the medical literature. While Kwon WJ et al. [14] reported that continuous ESP block offers effective pain management after mastectomy with sentinel/axillary lymph node dissection [15], Thiagarajan P et al. suggest that an ESP block may not be sufficient in covering the axillary region, resulting in the need for intraoperative fentanyl, like patients who did not receive an ESP block [16].

There are several benefits to using a catheter during surgery. It allows for adjusting the anaesthetic dose based on the patient's needs, extending the analgesic effect with an elastomeric pump, and repeating a bolus if necessary. For certain procedures, such as external quadrants and axillary lymph node biopsy or dissection, we typically use the BRILMA block. However, we have discovered that combining different fascial blocks and injecting an anaesthetic solution at various points on the same nerve results in better anaesthetic coverage. Using these blocks together has enabled us to avoid general anaesthesia in patients with multiple health conditions, who have a higher risk of complications, as noted by Bruceta M et al, including increased blood loss, infections, and postoperative respiratory insufficiency [17].

If we were to administer general anaesthesia and systemic opioid pain therapy to this patient, it would necessitate admission to the intensive care unit (ICU) for close monitoring of their vital signs. Additionally, their awakening would require management, and postoperative pain therapy would need to be administered. Research conducted by Bruceta M et al. using data from the National Surgical Quality Improvement Program (NSQIP) database shows a significant correlation between high BMI and ICU admission following surgery. This group of patients often has additional health conditions such as hypertension, diabetes, cardiovascular disease, and obstructive sleep apnea, which can make surgery more challenging and lead to increased complications [17].

In cases of obstructive sleep apnea (OSA), administering systemic opioids could lead to respiratory depression. To prevent this potential risk, we have chosen to utilize the ketodex sedation method, which combines ketamine and dexmedetomidine. Ketamine works by depressing the thalamus-cortical system and activating the limbic system, inducing dissociative anaesthesia. It also maintains breathing and airway reflexes, boosts blood pressure and heart rate, and encourages mild bronchodilation. Dexmedetomidine is a potent alpha-2 selective agonist that induces sedation by reducing activity in the locus coeruleus, without causing significant cardiovascular depressive effects like other alpha agonists (e.g., clonidine). Additionally, dexmedetomidine does not inhibit breathing.

In this case, the combination of ketamine and dexmedetomidine was used to balance out each other's side effects and provide several benefits such as hemodynamic stability, no respiratory depression, postoperative analgesia, and smooth recovery [18]. Dexmedetomidine helped prevent tachycardia and hypertension caused by ketamine, while ketamine prevented bradycardia and hypotension caused by dexmedetomidine.

Our priority was to maintain spontaneous breathing and airway reflexes while avoiding the use of opioids to achieve complete anaesthetic coverage of the operative site. Based on the information provided, we used an opioid-free anaesthesia (OFA) strategy by combining regional anaesthesia with ketodex sedation to reach an appropriate anaesthetic level. This procedure helped the patient recover without any pain or risk and resume normal work and daily activities promptly after the intervention.

New evidence suggests that OFA can lead to better results for patients who undergo surgery, particularly for those who are more susceptible to the negative effects of opioids [9]. OFA is a multi-faceted analgesic technique designed to avoid opioid tolerance. However, it has not been thoroughly described how much customization of OFA is possible and beneficial [19]. The primary reasons for utilizing OFA are in bariatric and oncological surgery, as well as for vulnerable patients who can benefit the most from this method [19]. However, there is a wide range of techniques and drugs available, making it impossible to consider OFA as a single technique [9]. A better understanding of nociception and its genetic and acquired factors would be an excellent starting point to pave the way for personalized OFA. This would help to maximize its feasibility in various contexts and increase its potential indications, as demonstrated in our experience.

## Conclusions

Perioperative planning for the management of multimorbid patients has been instrumental in designing the optimal anaesthetic strategy. In our case, the association of continuous ESP plus BRILMA blocks associated with completely opioid-free anaesthesia ensured the successful execution of breast surgery without complications in a patient with multimorbidity. Furthermore, the diligent intraoperative monitoring made it possible to conduct the operation with the patient maintaining spontaneous breathing, without the need for intubation, which would have posed challenges in airway management. In conclusion, a thorough assessment of the patient's condition enables the selection of the best possible personalized anaesthetic plan, ensuring the utmost satisfaction for both the patient and the medical team.
